# MAGE-A Antigens and Cancer Immunotherapy

**DOI:** 10.3389/fmed.2017.00018

**Published:** 2017-03-08

**Authors:** Paul Zajac, Elke Schultz-Thater, Luigi Tornillo, Charlotte Sadowski, Emanuele Trella, Chantal Mengus, Giandomenica Iezzi, Giulio C. Spagnoli

**Affiliations:** ^1^Oncology Surgery, Department of Biomedicine, University Hospital of Basel, Basel, Switzerland; ^2^Department of Pathology, University Hospital of Basel, Basel, Switzerland; ^3^Cancer Immunotherapy, Department of Biomedicine, University Hospital of Basel, Basel, Switzerland

**Keywords:** MAGE-A, cancer–testis antigens, cancer immunotherapy, clinical trials, adoptive immunotherapy

## Abstract

MAGE-A antigens are expressed in a variety of cancers of diverse histological origin and germinal cells. Due to their relatively high tumor specificity, they represent attractive targets for active specific and adoptive cancer immunotherapies. Here, we (i) review past and ongoing clinical studies targeting these antigens, (ii) analyze advantages and disadvantages of different therapeutic approaches, and (iii) discuss possible improvements in MAGE-A-specific immunotherapies.

## MAGE-A Tumor-Associated Antigens

MAGE-A were the first human tumor-associated antigens identified at the molecular level (1). They belong to the larger family of cancer/testis antigens (CTA), whose expression is consistently detected in cancers of different histological origin and germinal cells (2). The MAGE-A sub-family includes 12 highly homologous genes located on chromosome Xq28 (3, 4). Specific gene products have been identified by immunohistochemistry in cancers of different histological origin, including high percentages of non-small cell lung cancers (NSCLC), bladder cancers, esophageal and head and neck cancers, and sarcomas (5). These antigens are also frequently expressed in triple negative breast cancers (6), myeloma (7), and Reed–Sternberg cells (8) in Hodgkin’s disease, with the highest frequency being detected in synovial sarcoma (9). Among healthy tissues, the expression of specific members of the family has been observed in spermatogonia, placenta (10), and fetal ovary (11). However, recently, MAGE-A1 and -A12 genes have been shown to be expressed in CNS as well, as discussed below (12).

## Functional Aspects of MAGE-A Antigens

Preferential intracellular location may be different for different antigens, e.g., mostly cytoplasmic for MAGE-A1, -A3, and -A4, but mostly nuclear for MAGE-A10 (13–16).

Functions are still unclear, although different studies have associated MAGE-A2, -A3/6, and -A9 expression with pro-tumorigenic activities such as p53 dysregulation (17–19), enhanced tumor cell proliferation potential, or maintenance of a cancer-stem cell-like functional profile (20).

In a variety of tumors of different histological origin, a clear correlation between expression of MAGE-A antigens and poor prognosis has been observed. In this context, data on bladder cancer (21, 22), NSCLC (23, 24), head and neck cancers (25–27), and ovarian cancer (28, 29) have consistently been reported. Indeed, MAGE-A antigen expression, at the gene and protein level, has repeatedly been shown to be associated with widespread DNA demethylation frequently observed in advanced cancers. On the same line, it has been shown to be inducible by demethylating agents, including chemotherapeutic compounds widely used in cancer treatment such as 5-aza-2′-deoxycytidine (30, 31), thus realistically envisaging the possibility of treatments combining chemotherapy and specific vaccination (32).

## Immunogenicity of MAGE-A Antigens

Although peptides restricted by both HLA classes I and II have been identified (33), naturally occurring adaptive immune responses to MAGE-A antigens are usually characterized by a very low frequency of specific precursors (34) in both healthy donors and patients bearing cancers expressing them (35). However, responses to MAGE-A10 have been more frequently detected (36, 37). Responses in tumor-associated lymphocytes (TIL) have seldom been explored, but we have observed that MAGE-A10-specific CTL could be expanded from TIL infiltrating NSCLC displaying a high expression of the target antigen (38). On the other hand, CTL recognizing peptide motifs shared by multiple MAGE-A proteins may be generated from peripheral blood from patients and healthy donors (39). Most recently, tumor reactive CD8+ T cells, isolated based on their expression of activation marker (PD-1) from peripheral blood of melanoma patients, have been shown to relatively frequently target MAGE-A antigens (40).

## Clinical Trials Targeting MAGE-A Antigens

In the past 10 years (2006–2016), a total of 44 clinical trials could be identified in “https://clinicaltrials.gov” database using “MAGE-A” as keyword: a total of 16 phase 0 or I, 13 phase I/II, 13 phase II, and 2 phase III studies. Regarding immunogen formulations, 16 studies utilized entire proteins in the presence or absence of adjuvants (41, 42), 11 used peptides (43–45), 6 used mRNA-transfected DC (46, 47), 1 was based on tumor cell lysate-pulsed DC, 2 took advantage of recombinant viral vectors (48, 49), and more recently, 6 and 2 trials, respectively, have focused on adoptive treatments by using specific T cell receptor (TCR)-transduced T cells (12) or expanded CTL (50).

Efficacy clinical data published so far, from patients immunized in the context of the 15 larger studies (phase II or III, Table S1 in Supplementary Material) mainly using MAGE-A protein (*n* = 11), do not appear to support significant clinical effectiveness (51).

Of interest, a chronological analysis of these 44 studies clearly underlines a strategy shift in the most recent years. Indeed, in the past 4 years, among the (only) 10 clinical studies initiated and including MAGE-A as antigens, there are no phase II or III studies. Moreover, the majority of the phase I or I/II studies are based on adoptive cell transfer. This “shift” in MAGE-A translational research strategy clearly results from the combined effect of “protein/peptide” efficacy failure and from the confidence generated by new approaches focusing on personalized effector T-cell treatment. In addition, one should also mention the shift in target paradigm from classical TAA to neo-antigens also contributing to the decreased use of MAGE-A antigens.

## MAGE-A3 Protein as Immunogen

One of the most important clinical trials ever performed in MAGE-A cancer immunotherapy, involving thousands of patients with NSCLC, was focusing on the administration of recombinant MAGE-A3 protein together with adjuvants (52, 53). Despite promising initial data and the proven ability of the immunization protocol to induce detectable humoral responses in vaccinated patients (54), disease-free interval in patients with completely resected stage IB, II, and IIIA NSCLC did not appear to be significantly prolonged, as compared to patients of control group, in phase III studies in the context of an adjuvant therapy setting (41).

Why did these trials fail to reach efficacy? First, similar to MAGE-A antigens, a large majority of classical TAA-specific cancer vaccines clinically tested so far have been shown to induce heterogeneous immune responses rarely resulting in significant clinical effects.

**Figure 1:**
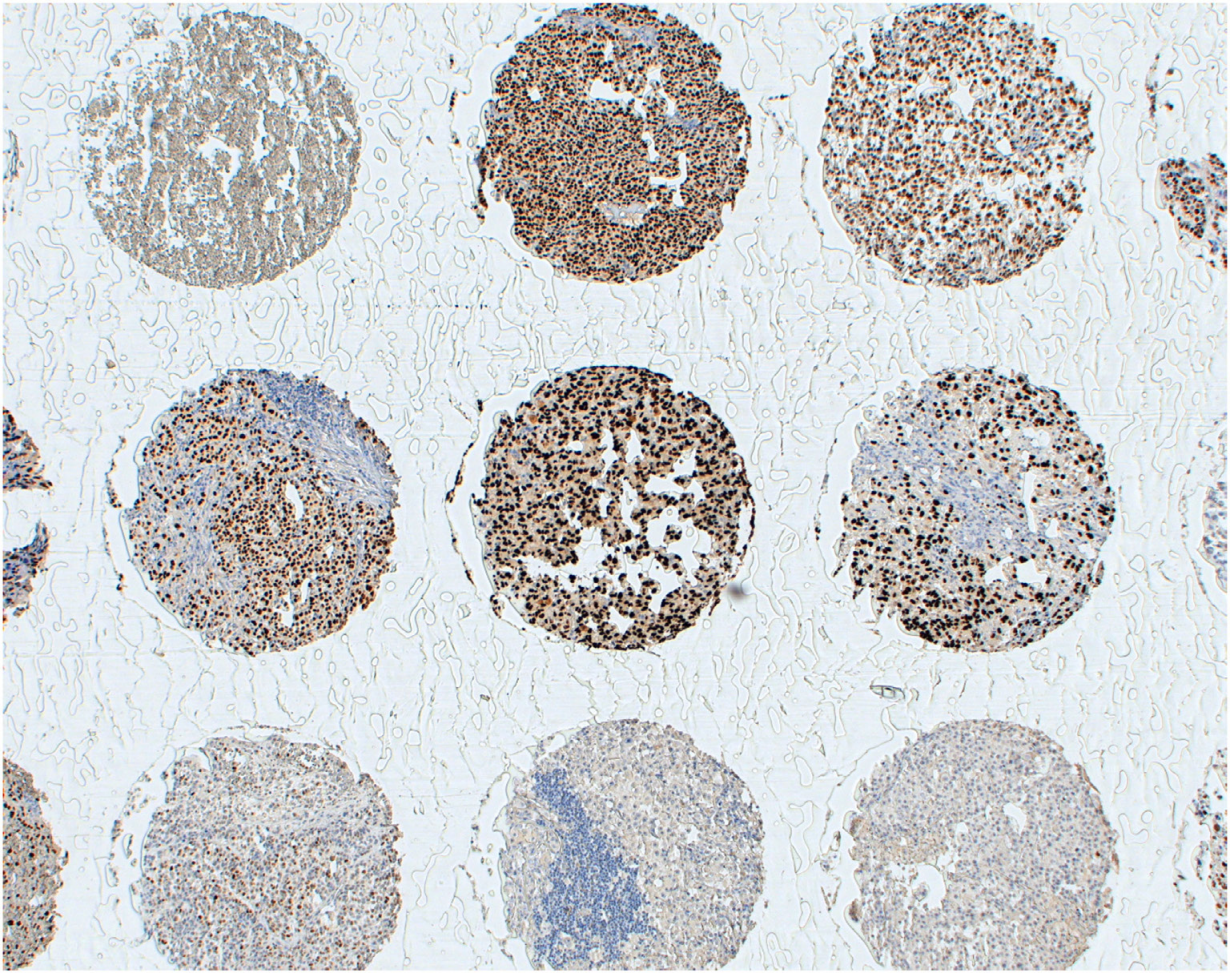
**Heterogeneity of MAGE-A10 expression at the protein level**. Melanoma tissues from a multi-tumor tissue microarray were stained with a MAGE-A10-specific reagent by standard techniques, as previously detailed (16). Antigen expression displays a high heterogeneity, regarding both percentages of antigen-positive tumor cells and staining intensity.

However, specific issues should be considered for CTA-specific immunization. For instance, MAGE-A CTA expression, a pre-requisite for the eligibility of patients for treatment in these studies, has usually been assessed at the gene level by quantitative RT-PCR (RT-qPCR) (41, 54), which cannot provide insights into the actual numbers of CTA-positive tumor cells. Immunohistochemical studies using available MAGE-A-specific mAbs consistently underline that expression of these antigens might be highly heterogeneous in cancerous tissues with high expression often only detectable in relatively low percentages of tumor cells (10, 55). Remarkably, due to the high homology of sequences from different components of the MAGE-A family, a majority of currently available reagents do recognize multiple antigens. Our own experience based on the use of a MAGE-A10 highly specific mAb (Figure 1) suggests that expression of these antigens may be highly heterogeneous in a variety of tumors of different histological origin, with percentages of “positive” cells ranging between 5 and >60% (16). One could speculate that criteria based on the expression of target antigen(s), at the protein level, in high percentages of tumor cells and in multiple areas of primary and metastatic cancers could be applied for a more stringent selection of patients potentially eligible for MAGE-A-targeted antitumor immunization. Additionally, it might be of interest to verify the expression of the target MAGE-A antigen in recurrent tumors following specific immunization protocols, to verify possible selective immune editing (56). It is worth noting, however, that successful antigen-specific vaccination has also been shown to be able to promote responsiveness against unrelated antigens, the so-called “antigen spreading” phenomenon (57), thus potentially overcoming the requirement for a uniform expression of target antigens in tumors to be treated.

Importantly, the recombinant protein used in most efficacy studies was shown to induce humoral response and HLA class II-restricted lymphoproliferation, as expectable (41, 53, 54). However, the ability of these antigen formulations to promote class I-restricted responses appears to be more limited. One could speculate that libraries of overlapping “long” peptides (58), or highly immunogenic recombinant vectors (38, 59), could be more effective in this regard.

## Heterogeneous Expression of MAGE-A Genes in Primary and Metastatic Cancers

Studies from our group clearly document the heterogeneity of MAGE-A antigens expression at the gene expression level as well. We tested by RT-qPCR the expression of Mage-A1, -A2, -A3, -A4, -A10, and -A12 genes in primary NSCLC from 33 patients (Table 1). In keeping with published data (23, 24), a total of 22 tumors (66%) showed evidence of expression of at least one of the antigens under investigation. Similar to recently published data in oral cancer (60), out of these patients with MAGE-A+ NSCLC, 10 (45%) had lymph nodes (LN) showing evidence of tumor metastasis, as compared with only 2 (18%) from the 11 MAGE-A(−) primary tumors. Interestingly, among the 10 metastatic LN from MAGE-A+ primary cancers, only half showed evidence of MAGE-A gene expression. Furthermore, in four LN, classified as non-metastatic, based on pathological evidence, expression of MAGE-A genes could be observed by RT-qPCR. Intriguingly, among LN associated with MAGE-A− primary cancers, 1/2 and 1/8 metastatic and non-metastatic samples, respectively, showed evidence of MAGE-A gene expression.

**Table 1:** **MAGE-A gene expression, as detected by RT-qPCR in primary non-small cell lung cancers (NSCLC) and in corresponding lymph nodes (LN) showing evidence of metastatic outgrowth by standard clinical pathology techniques**.

Total number of patient	1 MAGE-A + RT-qPCR	2 LN-met histo	3 LN-MAGE-A + RT-qPCR
33	22+	10+	5+
			5−
		12−	4+
			8−
	11−	2+	1+
			1−
		9−	1+
			8−

*Tissues obtained from surgical resections from patients with NSCLC were tested by RT-qPCR for Mage-A1, -A2, -A3, -A4, -A10, and -A12 gene expression. Positivity (+) was defined by expression of at least one target gene above threshold (threshold = delta Ct to β-actin < 10). LN were similarly assessed by RT-qPCR and standard clinical pathology scoring*.

Taken together, these data suggest a higher sensitivity of RT-qPCR as compared to standard techniques for the detection of cancer cells within LN draining primary tumor tissues. Most importantly, however, they confirm the dynamic nature of MAGE-A antigens expression during cancer progression and may support the concept of combination therapies including treatments promoting MAGE-A antigen expression together with specific immunization procedures (61).

## Adoptive Immunotherapies

In recent clinical studies, effector T cells, transduced with vectors encoding for specific TCRs recognizing peptides from MAGE-A3 or MAGE-4, have been adoptively transferred into patients bearing tumors expressing these antigens. Unfortunately, upon anti-MAGE-A3, HLA-A0201-restricted TCR gene therapy, despite measurable clinical responses in some patients, treatment-related severe adverse events and deaths were also reported. These events may possibly be due to the high affinity of these TCRs (see below) and to the recognition (“on-target/off-tumor”) of highly homologous peptide(s) from other MAGE-A proteins expressed in the CNS (12, 62). Similarly, myocardial toxicity, resulting in treatment related death, has also been observed following gene therapy with a MAGE-A3-specific HLA-A0101-restricted TCR (63, 64). In the latter case, the “off-target” effect was attributed to the high homology between the target peptide and a peptide from Titin muscle protein.

It is worth noting that the TCR transduced into T cells in the first study originally derived from “humanized” mice expressing HLA-A0201 and its affinity toward the target antigen was further improved by site-directed mutagenesis (65), thus increasing the chances of “on-target_off-tumor” adverse events affecting tissues characterized by low but detectable expression of defined MAGE-A antigens (12). The affinity of the TCR used in the second study, originally derived from a patient immunized with a recombinant viral vector (66), was also enhanced by site-directed mutagenesis.

By contrast, T cells expressing a MAGE-A4-specific TCR have been safely used in adoptive immunotherapy of patients with recurrent esophageal cancer (67).

Taken together, these data suggest that the clinical use of enhanced TCR effectors targeting MAGE-A antigens for cancer immunotherapy should be carefully evaluated in order to minimize potential “off-tumor” side effects.

However, natural MAGE-A-specific TCRs, from clones derived from tumor bearing patients or healthy donors, might also be of interest. Such CTLs would probably be characterized by a lower affinity for cognate HLA–class I peptide complex and possibly by a lower antitumor effector potential, but they would also likely have less toxic side effects. Considering the cumulative potency related to the high numbers of transduced cells usually infused into treated patients, and their ability to proliferate and generate “memory,” the effectiveness of this type of treatment should reasonably be further tested.

## Conclusions

Taken together, published data may suggest that therapeutic strategies targeting MAGE-A antigens have so far failed to fulfill the promise of representing effective tools for cancer treatment. However, the understanding of mechanisms controlling immune response as a whole and cancer-specific immune responses in the tumor microenvironment in particular has made enormous progress in the past decade, generating an unprecedented “momentum” for cancer immunotherapy.

Successful utilization of therapeutic mAbs recognizing “immunological checkpoints” is currently generating enormous interest in clinical oncology. Their mechanisms of actions (MoA) are not fully clarified (68, 69). However, one of the main MoA is arguably represented by the “release of brakes” hampering T cell responses specific for tumor-specific or associated antigens. This hypothesis is supported, for instance, by the higher effectiveness of treatment with anti CTLA-4 therapeutic mAbs in cancers characterized by a high mutational load, likely to result in a higher expression of mutated proteins potentially recognized as “non-self” by the adaptive immune system (70). It is therefore reasonable to postulate that adequately timed combinations of vaccination procedures and administrations of therapeutic “checkpoint inhibitor” specific mAbs could be of high clinical relevance. Within this framework, a critical point might be represented by the choice of antigens of potential clinical use. Neo-antigens, e.g., tumor-specific mutated proteins have been successfully identified by whole exome sequencing (71–73), and the expression of defined antigenic “non-self” peptides associated with restricting HLA class I determinants may be detected by mass spectrometry techniques (74). Although highly appealing, the “personal” nature of neo-antigens might possibly also represent their Achilles’ heel, not only because of regulatory hurdles (75) but also because it would likely prevent the performance of conventional randomized trials, thereby complicating a reliable assessment of the effectiveness of innovative treatment procedures.

Based on these considerations, vaccination with tumor-associated or CTA could still realistically find an important place in cancer immunotherapy in the era of “immunological checkpoint” inhibitors (76). Considering that MAGE-A antigens are expressed in tumors with poor prognosis and a scarcity of therapeutic options, such as TNB, and lung and esophageal cancers, it is easy to predict that the interest of the scientific community in CTA might actually be revived in the light of the enormous advances in cancer immunotherapy of the last years.

## Author Contributions

All authors participated in writing the manuscript and/or revising it critically for important intellectual content or providing the data mentioned in the manuscript.

## Conflict of Interest Statement

The authors declare that the research was conducted in the absence of any commercial or financial relationships that could be construed as a potential conflict of interest.
