# Supplementary material for: MAGE-A Antigens and Cancer Immunotherapy
Source: Front Med (Lausanne). 2017 Mar 8;4:18. doi: 10.3389/fmed.2017.00018 (PMC5340762; doi:10.3389/fmed.2017.00018)
Supplement: Supplementary file 1 [file table_1.pdf]

Supplementary Table

NCT identif.	phase 2 or 3	start date	status (end 2016)	cancer type	MAGE-A included	formulation	Nb of patient	info	Survival	Ab	T-cell
NCT00480025(1)	Phase 3	Oct-07	Terminated	NSC Lung Cancer	MAGE-A3	protein	2278	MAGRIT	no sign. diff/placebo	100%	n.a.
NCT00796445(2)	Phase 3	Dec-08	Terminated	Melanoma	MAGE-A3	protein	1351			99%	n.a.
NCT00896480(3)	Phase 2	May-09	Completed	Melanoma	MAGE-A3	protein	24	"gene signature" (GS+/GS-) grouping trials	no diff GS+/GS-	100%	15/17 CD4+ 3/17 CD8+
NCT00849875(4)	Phase 2	May-09	Early Terminated	Melanoma	MAGE-A3	protein	48			100%	n.a.
NCT00942162(5)	Phase 2	Aug-09	Completed	Melanoma	MAGE-A3	protein	125			100%	n.a.
NCT01159288(6)	Phase 2	Dec-09	Unknown	NSC Lung Cancer	MAGE-A1+A3	Peptide	47	DC exosome	no signif. results	no	2/8 CD8+
NCT00960752	Phase 2	May-10	Active, not recruiting	Melanoma	MAGE-A3	Peptide	71	+ Resiquimod	No result available		
NCT01245673(7)	Phase 2	Nov-10	Active, not recruiting	Myeloma	MAGE-A3	Peptide classI+II	28	+ Poly-ICLC +adopt Tcell	74% /2y OS	78% (+adj)	76%
NCT01302496(8)	Phase 2	Feb-11	Active, not recruiting	Melanoma	MAGE-A3 (+C2)	mRNA-DC (class-II)	39	+ ipilimumab	51% DCR/6mo. 8CR 7PR	n.a.	1/10 MAGE-C2-CTL
NCT01435356	Phase 2	Aug-11	Active, not recruiting	Bladder	MAGE-A3	protein	273	MAGNOLIA	No result available		
NCT01437605	Phase 2	Oct-11	Active, not recruiting	Melanoma	MAGE-A3	protein	14	+ Poly-ICLC	No result available		
NCT01266603(9)	Phase 2	Feb-12	Active, not recruiting	Melanoma	MAGE-A3	protein	30	+ IL2	4/16 PR	n.a.	n.a.
NCT00373217	Phase 2	Feb-08	Early Terminated	Fallopian tube, Ovarian, Perit. cav.	MAGE-A1	Peptide	6		No result available		
NCT00706238	Phase 2	Sep-08	Early Terminated	Melanoma	MAGE-A3	protein	6		No result available		
NCT01307618	Phase 2	Feb-11	Early Terminated	Melanoma	MAGE-A3	protein	10		No result available		

Table S1: List of clinical trial initiated within the past 10 years, addressing clinical efficacy (phase 2 or 3) and including at least one MAGE-A antigen in the immunogen formulation.

Reference List

1. Vansteenkiste JF, Cho BC, Vanakesa T, et al. Efficacy of the MAGE-A3 cancer immunotherapeutic as adjuvant therapy in patients with resected MAGE-A3-positive non-small-cell lung cancer (MAGRIT): a randomised, double-blind, placebo-controlled, phase 3 trial. *Lancet Oncol* 2016;**17**(6): 822-835.

2. A double-blind, randomized, placebo-controlled Phase III study to assess the efficacy of recMAGE-A3 + AS15 ASCI as adjuvant therapy in patients with MAGE-A3 positive resected stage III melanoma. https://www.clinicaltrialsregister.eu/ctr-search/trial/2008-002447-16/results, NCT00796445, 15-10-2016

3. An open Phase II study to assess the clinical activity and safety of recMAGE-A3 + AS15 cancer immunotherapeutic in patients with metastatic cutaneous melanoma, and to explore its immunogenic properties, including their relation to tumor infiltration, genomics and proteomics https://www.clinicaltrialsregister.eu/ctr-search/trial/2008-001301-42/results, NCT00896480, 3-3-2016

4. An open Phase I/II study of immunization with the recMAGE-A3 + AS15 Antigen Specific Cancer Immunotherapeutic in association with dacarbazine in patients with MAGE-A3 positive unresectable and progressive metastatic cutaneous melanoma https://www.clinicaltrialsregister.eu/ctr-search/trial/2008-001918-25/results, NCT00849875, 13-2-2017

5. Saiag P, Gutzmer R, Ascierto PA, et al. Prospective assessment of a gene signature potentially predictive of clinical benefit in metastatic melanoma patients following MAGE-A3 immunotherapeutic (PREDICT). *Ann Oncol* 2016;**27**(10): 1947-1953.

6. Besse B, Charrier M, Lapierre V, et al. Dendritic cell-derived exosomes as maintenance immunotherapy after first line chemotherapy in NSCLC. *Oncoimmunology* 2016;**5**(4): e1071008.

7. Rapoport AP, Aqui NA, Stadtmauer EA, et al. Combination immunotherapy after ASCT for multiple myeloma using MAGE-A3/Poly-ICLC immunizations followed by adoptive transfer of vaccine-primed and costimulated autologous T cells. *Clin Cancer Res* 2014;**20**(5): 1355-1365.

8. Wilgenhof S, Corthals J, Heirman C, et al. Phase II Study of Autologous Monocyte-Derived mRNA Electroporated Dendritic Cells (TriMixDC-MEL) Plus Ipilimumab in Patients With Pretreated Advanced Melanoma. *J Clin Oncol* 2016;**34**(12): 1330-1338.

9. McQuade JL, Torres-Cabala CA, Murthy R, et al. A phase II trial of high-dose Interleukin-2 (HDIL-2) with recombinant MAGE-A3 protein combined with adjuvant system AS15 in patients with unresectable or metastatic melanoma. *Cancer Research* 2016;**76 (suppl)**(14 suppl).
